# Policy options for sustainable access to off-patent antibiotics in Europe

**DOI:** 10.1038/s44259-024-00061-4

**Published:** 2024-11-25

**Authors:** Dimitra Panteli, Michael Anderson, Thomas Fieldman, Enrico Baraldi, Thomas Tängdén, Sabine Vogler, Christine Årdal, Elias Mossialos

**Affiliations:** 1 European Observatory on Health Systems and Policies, Brussels, Belgium; 2 Health Organisation, Policy, Economics (HOPE), Centre for Primary Care & Health Services Research, The University of Manchester, Manchester, UK; 3 LSE Health, Department of Health Policy, London School of Economics and Political Science, London, UK; 4 Department of Clinical Microbiology, Cambridge University Hospitals NHS Foundation Trust, Cambridge, UK; 5 Department of Business Studies, Uppsala University, Uppsala, Sweden; 6 Department of Medical Sciences, Uppsala University, Uppsala, Sweden; 7 WHO Collaborating Centre for Pharmaceutical Pricing and Reimbursement Policies, Pharmacoeconomics Department, Gesundheit Österreich GmbH (GÖG / Austrian National Public Health Institute), Vienna, Austria; 8 Department of Health Care Management, Technische Universität Berlin, Berlin, Germany; 9 Antimicrobial Resistance Centre, Norwegian Institute of Public Health, Oslo, Norway

**Keywords:** Health policy, Health services, Health care economics, Drug regulation

## Abstract

Securing sustainable access to existing antibiotics optimises agent choice for individual treatments and is crucial to curb antibiotic resistance. Access to antibiotics is often restricted in many countries, due to general market unavailability or episodic shortages. This article outlines key policy options to maintain availability of existing antibiotics and enhance antibiotic supply chain resilience focusing on the perspectives of European Union (EU) and European Economic Area (EEA) institutions and member states.

## Introduction

Antimicrobial resistance (AMR) poses a significant challenge for health systems worldwide^1^. One driver of AMR, and antibiotic resistance in particular, is lack of access to first-line antibiotics for optimal treatment^2^. This lack of access impairs the management of primary infections and undermines potentially life-saving interventions in areas such as surgery and oncology, while also accelerating resistance development. For example, the substitution of broader spectrum or suboptimal agents for unavailable narrow spectrum agents may increase resistance^3,4^. While research and development into new antibiotic agents is essential to diversifying the anti-infection arsenal^5^, it is equally important that access to existing, largely off-patent antibiotics is improved. Indeed, the global death rate from lack of antibiotic access is greater than that from AMR^6^.

Most countries experience shortages of off-patent medicines, including antibiotics. Shortages are defined as supply not meeting demand at the national level^7^. In 2019, countries in the European Union (EU) issued over 1300 notifications of antibiotic shortages^8^. Such shortages can lead to increased use of broad-spectrum antibiotics and potential long-term negative effects, such as physicians changing their prescribing habits and reducing compliance with evidence-informed prescribing guidelines^9,10^. Patients may be directly harmed if lack of access to the most effective antibiotics necessitates substitution with less effective agents^10^. Moreover, shortages of antibiotics can also create market opportunities for substandard or falsified products^11,12^.

Supply chain insecurity is generally due to the small number and geographic concentration of actors responsible for key stages in the antibiotic supply chain, including manufacture of active pharmaceutical ingredients (APIs) and conversion of APIs to medicines^13^. A global analysis of 40 antibiotics for both human and veterinary use found that nearly 70% of antibiotic API manufacturing sites are located in India (35%) and China (34%)^14^. China is also the largest exporter of antibiotic APIs, accounting for 71% (of kilotons of product) of international exports in 2020. Many off-patent antibiotics for human and veterinary use are produced using single source APIs, and the global supply of many essential antibiotics for both humans and animals, such as cephalosporins, macrolides and penicillin, depends on a few key API suppliers in China^15^. Data sources do not distinguish between human and veterinary markets; however, given that livestock consume an estimated 70% of all antibiotics and require higher amounts of APIs, these figures should be viewed with caution^16^. Yet it is clear that the global antibiotic supply is vulnerable to disruptions affecting even a small number of actors, such as accidents or trade restrictions^10,17^. The international and prolonged shortage of piperacillin-tazobactam in 2017 following an explosion in a Chinese factory that was responsible for producing most of the world’s supply of the API necessary for its production clearly demonstrates this reality^10^.

Challenges in forecasting antibiotic demand and monitoring supply further contribute to shortages. These include commercially sensitive information leading to lack of transparency extending from manufacturers and market-authorization holders (MAHs) to relationships with API suppliers^18^. Additionally, there is a responsibility vacuum at both national and international levels surrounding the task of balancing demand and supply of antibiotics in a continuous, consistent manner.

In addition to episodic shortages, the limited availability of antibiotics may result from MAHs withdrawing their products from or not entering certain markets^18–20^. Comparisons between countries show considerable variability in the number and range of antibiotic products available^20–23^. Financial considerations, particularly the expectation of insufficient revenue, are cited as the main reason for MAHs withdrawing or not launching antibiotics^24^. This is driven by relatively small expected volumes (given epidemiological trends and conservation efforts), brief treatment regimens and low prices (especially for older antibiotics with generic competition). Lack of clinician demand and non-inclusion in national prescribing guidelines also contribute to the problem, although efforts exist in some European countries to accelerate adoption of updated antibiotic guidelines^25,26^. While lobbying by professional groups may restore availability of older “forgotten” antibiotics, such as temocillin and amikacin^22,27^, a systematic approach to ensuring these medicines remain on the market is lacking.

In this article, we discuss a range of selected policy options for improving access to existing antibiotics for human health, with a particular focus on the EU context. Procurement of medicines, including antibiotics, is a competence of the EU member states; however, the EU can complement member state policies to address common challenges, such as AMR. At the same time, the European Medicines Agency (EMA) is not only responsible for the central authorisation of medicines in the EU (including most new antibiotics since the establishment of this procedure in 2005) and providing the relevant scientific guidelines, but also collects data on antimicrobial consumption in animals, and - as per its reinforced mandate under the European Health Union – monitors medicines shortages and takes an active role in addressing them^28^. The Health Emergency Preparedness and Response Authority (HERA), established in September 2021 to strengthen preparedness planning and response across the EU^29^, has a mandate to enhance stockpiling capacity of medical countermeasures and is currently developing a stockpiling strategy, known as the rescEU reserve^30^. It also hosts the Critical Medicines Alliance (CMA), a multi-stakeholder consultative mechanism set up in January 2024 that aims to identify key priorities for action and propose solutions to strengthen the supply of critical medicines in the EU towards addressing shortages.

This article focuses on off-patent antibiotics, which are not subject to patent protection and other exclusivities and are thus eligible for generic production and market entry. Challenges to improving access to newer antibiotics in the EU have been mapped elsewhere^5^. Our selection and grouping of policy options is inspired by the Swedish multisectoral collaboration PLATINEA (Platform for Innovation of Existing Antibiotics), which includes 21 stakeholders from academia, healthcare, industry and public agencies who systematically discuss the causes of, and potential solutions to, antibiotic shortages^31^, and which has identified the underlying causes of shortages, including excessive costs, insufficient revenues for suppliers and short- and long-term supply chain weaknesses. We also draw on work carried out by the European Observatory on Health System and Policies in the context of the 2023 Swedish Presidency of the Council of the EU to outline policy options to improve access to new and existing antibiotics^32^. Each exercise leveraged groups of experts, who met to discuss relevant challenges to improving access to antibiotics and the potential benefits, limitations and operational feasibility of different policy options.

We do not describe each measure in detail; rather, our contribution lies in the coherent, thematic grouping of measures into the building blocks of a multi-component approach to improve access to existing antibiotics. While we focus on operational feasibility within the EU, this approach and the individual measures described are applicable to other contexts.

### Policy option categories

We discuss four groups of policy options to strengthen antibiotic availability: (1) administrative and regulatory levers; (2) purchasing arrangements (both of these groups can mitigate commercial unattractiveness by reducing costs or increasing revenues for MAHs); (3) product mapping and stockpiling to improve short-term availability in specific sections of the supply chain but which may have negative knock-on effects; and (4) manufacturing capacity mapping and strengthening to dynamically and proactively improve supply in the long-term. Table 1 summarises the proposed options and their operational feasibility in the EU.

**Table 1:** Selected policy options for improving access to existing antibiotics

Policy domains	Specific policies	Definition	Operational feasibility within the EU/EEA
Administrative and regulatory levers	Reduce or waive marketing authorisation fees	Reduce or remove administrative fees needed for regulatory permission to put antibiotics on the market.	These measures, levied by national regulatory authorities and potentially by the European Medicines Agency (EMA), may require investment trade-offs, such as fee adjustments for other medications. They also entail the risk of “encouraging” requests for exemption from other therapeutic classes.
	Adapt sunset clauses	Modify or remove market authorisation “sunset clauses” activated by EU or national laws for medications previously on the market but absent for three consecutive years^33^.	EMA or national regulators would need to establish that there are no safety or efficacy concerns for antibiotics absent from the market for more than three years. Implementation would need to include clear eligibility criteria that antibiotics meet important public health needs and ongoing monitoring of their efficacy and safety.
Purchasing arrangements	Ensure good procurement practice/Change tendering contracts	Develop tendering contracts that stipulate multiple suppliers and favour at least one European manufacturer.	This approach would require some changes to member state procurement guidelines and regulatory frameworks. The successful Nordic precedent provides insights into good practices for engaging multiple suppliers. However, recent German experience highlights potential challenges when European manufacturers are lacking^68^.
	Joint procurement	Collectively publish joint tenders through multiple buyer (such as multiple countries) collaboration.	Several EU/EEA cross-country collaborative joint tenders, such as the Nordic Pharmaceutical Forum, prove the feasibility of joint procurement of off-patent antibiotics^45^. This approach is also feasible at the EU level and could utilize the EU Joint Procurement Agreement or other instruments.
	Subscription payments	Counteract financial unattractiveness by basing antibiotics payments on contractually agreed annual revenues that may be independent of (‘delinked’ from) sales volume.	Implementing this approach would require political will and consideration of the differing payment abilities of member states. Sweden’s annual revenue guarantee pilot, which partially delinks revenue from sales and includes the older antibiotic fosfomycin for intravenous administration, was evaluated positively^50^. Numerous novel medicine subscription-fee examples, such as the UK (novel antibiotics)^74^, Australia (new Hepatitis C medicines)^75^, and the US (new Hepatitis C medicines)^75^, provide inspiration.
Harmonisation and stockpiling of products	Harmonise strengths and formulations	Identify opportunities to harmonize strengths and formulations of antibiotics across Europe to minimise unnecessary differences lacking clear clinical rationale.	This would require mapping antibiotic strengths, formulations and package sizes in EU/EEA countries to identify opportunities for harmonization. The EMA has begun this process for paediatric doses and formulations of amoxicillin^52^. Formalising antibiotic harmonization would require significant collaboration with national procurement agencies and generic manufacturers potentially reluctant to share commercially sensitive information. Engagement and buy-in from professional societies would be required to adjust national prescribing guidelines.
	Harmonise packaging and labelling	Design appropriate packaging and labelling for users in multiple EU countries	Implementing this may be easier regionally (e.g., for groups of countries, such as the Nordics)^19^, but also potentially feasible at the EU level (currently under discussion)^54^. This is particularly relevant in hospital settings, where clinicians may either use English language packaging or an electronic version in the local language. The revisions to the EU’s pharmaceutical legislation proposed in April 2023 recognise the potential of multi-language packaging for improving access in smaller markets and include the possibility for Member States to include relevant provisions (see Article 74 of the proposal for a Directive on the Union Code relating to medicinal products for human use)^76^.
	Create physical stockpiles	Procure and store physical stockpiles of medications through public funding or mandatory stockpiling by suppliers.	HERA’s mandate explicitly includes increasing the stockpiling capacity of medical countermeasures, including through the use of EU4 Health funds^29^. This initiative could utilize the European resources reserve (rescEU reserve). Physical stockpiles can also be promoted through additional financing for mandatory private sector inventory increases. Stockpiles should be designed to ensure they do not create scarcity in less well-resourced countries.
	Create virtual stockpiles	Facilitate the exchange of supply, surplus and global shortage information by creating and maintaining a virtual data warehouse of EU/EEA antibiotic needs and deployable stocks^77^.	This could build on existing systems such as the European Shortages Monitoring Platform (ESMP) or the European Medicines Verification System (EMVS).
	Lending agreements	Devise agreements between countries to lend stocks of antibiotics when shortages arise.	This requires that countries have sufficient antibiotic stockpiles to redistribute to other countries. This may not be feasible if there is a simultaneous surge in demand across multiple countries. However, the Baltic Procurement Initiative has demonstrated that this approach can work for both medicines and medical devices^44^.
Strengthening manufacturing capacity	Map production capacity	Identify distribution of suppliers capable of producing antibiotics or their precursors, and their ability to increase output if required.	This may require significant collaboration with national agencies and generic manufacturers reluctant to share commercially sensitive information. A recent feasibility study identified potential measures to increase information transparency in the event of shortages, but noted that supply chain setup information is often protected and difficult to obtain^54,65^. However, New Zealand serves as an example, where the Medicines and Medical Devices Safety Authority provides publicly available information on names and locations of active pharmaceutical ingredient producers, finished product manufacturers, product sponsors and product marketers^67^.
	Invest capital in manufacturing capacity	Use resources to develop antibiotics production capability (APIs and finished medicines) in the EU.	Although this could be achieved via subsidies, it would require careful consideration of their nature and the implications for competition in the common market, as well the limitations imposed by state aid rules.

### Administrative and regulatory levers

MAHs may withdraw their products if annual registration fees exceed expected product revenues; administrative costs may apply for re-entering the market. When MAHs abandon markets without the active withdrawal of marketing authorisations, national regulators may invoke so-called “sunset clauses” as per national or EU laws, in the expectation of the availability of sufficient volumes^33^. Sunset clauses result in the cessation of a medicine´s marketing authorisation after a defined period of product unavailability, potentially creating financial and administrative barriers to reauthorisation and thus limiting access to older antibiotics.

Potential regulatory policies to discourage the withdrawal of existing antibiotics include reducing or waiving annual fees and adapting sunset clause requirements. This approach has already been implemented in Norway and suggested in Sweden^19^. At the EU level, similar policies might be applied to antibiotics via central marketing authorisation by the EMA. In April 2023, the European Commission proposed a comprehensive package of revisions to the existing pharmaceutical legislative framework, including the abolition of sunset clauses^34^. At the national level, member states could also consider abolishing sunset clauses for those generic antibiotics which received regulatory approval prior to the formation of the EMA in 1995.

These regulatory levers could apply to all antibiotics or could be combined with clear criteria for exemption from annual fees and sunset clauses, such as public health importance (e.g., linked to expected effectiveness and extent of clinical applications in real-world settings) and expected sales values. However, waiving annual fees for antibiotics may require adjusting other medication fees to ensure the financial stability of regulatory agencies. Other medicines important for public health might also merit exemption considerations and antibiotics waivers could be perceived as a precedent, encouraging other medicine MAHs to request similar exemptions.

Other options to lower the threshold for reauthorisation may include allowing authorisation based on older documentation and fast-tracking reauthorisation processes for antibiotics^19^. Public authorities might consider proactively engaging with manufacturers to discuss or signal a desire for reauthorisation. For example, in 2023, when Norway increased the unit prices of select antibiotics, it included many products that had previously abandoned the market^35^.

These measures require relatively low-threshold changes to improve sustained accessibility to existing antibiotics. However, in isolation, their effectiveness in counteracting current market failures would most likely be limited. These measures reduce some MAH-borne costs, namely those directly related to market access, but they do not directly affect the other main financial variable, i.e., the size of expected sales revenue. This issue is addressed by the next group of policies.

### Purchasing arrangements

Pharmaceutical procurement focuses predominantly on unit price, despite EU procurement law encouraging procurers to apply multiple award criteria^36^, following the Most Economically Advantageous Tender (MEAT) approach. Prices for off-patent medicines are generally low due to automatic price reductions, lowest-price-based tenders and reimbursement policies that favour the lowest-priced medicines within a comparable cluster. These low prices often mean that suppliers may not always have the capacity to remain financially viable when entering contracts to supply off-patent antibiotics. Increasing the attractiveness of antibiotic markets solely by raising unit prices is challenging, owing to the modest size of target populations, short treatment durations and antibiotic conservation efforts. Therefore, approaches that increase sales volume predictability or delink it from revenue merit consideration^37^.

A general prerequisite for any approach to purchasing is understanding and applying good procurement practices, including contracting more than one supplier to avoid race-to-the-bottom tendering and devising contracts of sufficient duration and sufficient minimum orders to attract adequate bids and improve predictability. Supply security should also be either a criterion for awarding contracts or a bonus reimbursement component when targets have been achieved. In the case of antibiotics, incorporating environmental criteria into procurement terms merits consideration both in terms of the One Health approach needed to tackle AMR and the responsibility to reduce the environmental footprint of healthcare as a whole. Examples of good European procurement practices have been mapped, and cross-country collaboration has been identified as a major proponent for their adoption^38^. The exchange of good procurement practices across countries can be facilitated through existing initiatives, such as the Network of Competent Authorities on Pricing & Reimbursement (NCAPR)^39^, an informal cooperation platform steered by the European Commission.

One option to improve the commercial viability of existing antibiotics, particularly those vulnerable to shortages, is for countries to increase market size through joint purchasing. In theory, joint procurement can enhance transparency through better information sharing, enable experience sharing, strengthen bargaining power and mitigate high transaction costs by pooling skills, capacities and negotiations. It can also ensure sustainable access to health technologies through cross-border exchange of products in short supply and can achieve sustainable prices through economies of scale, thereby improving the quality of purchased goods, ensuring supply security and availability, and fostering innovation^40^. Since 2020, Denmark, Iceland and Norway have pooled tendering for hospital antibiotics with non-price attributes, including surety of supply and environmental considerations, comprising 75% of award criteria. As a result, withdrawn antibiotics have re-entered these markets^41^. Successful implementation of such initiatives requires political will and trade-off flexibility^38,40^.

At the EU level, joint purchasing of antibiotics could be conducted within the scope of the EU Joint Procurement Agreement (JPA) which applies to medical countermeasures addressing serious cross-border threats to health^42^. Motivated by the experience of the 2009 H1N1 pandemic, the JPA was adopted in 2014 to improve member state purchasing power and strengthen solidarity. Some JPA provisions were amended in light of the COVID-19 pandemic (see Article 12 of Regulation 2022/2371)^43^, including the introduction of a potential exclusivity clause to prevent participating countries from running parallel negotiation processes and procuring medical countermeasures through other channels. Other voluntary cross-country collaborations for pharmaceutical and vaccine procurement can also provide lessons for potential joint antibiotic procurement efforts^44^.

Regional joint procurement mechanisms within the EU, such as the Nordic Pharmaceutical Forum^45^, or the Baltic Procurement Initiative, provide potential platforms for joint procurement of antibiotics between countries^44^. International examples of joint procurement mechanisms, including initiatives among African Island states^46^, the Gulf Cooperation Council (GCC)^47^, and the Pan-American Health Organization (PAHO) Revolving Fund (RF)^48^, could be applied to older antibiotics. There are also existing international partnerships that could facilitate joint procurement of antibiotics, such as the Global Antibiotic Research & Development Partnership (GARDP) and the Stop TB Partnership’s Global Drug Facility (GDF)^49^.

To maximise gains and prevent overcentralisation, participating countries would need to agree on a priority list of jointly-purchased antibiotic products. However, a shift towards more centralised processes could reduce opportunities for local MAHs to win (central) tenders, thus endangering their sustainability and jeopardising supply chain diversification.

Further steps to partially or fully delink revenue from sales volume and prices can also be considered. Subscription-style antibiotic payment models hold promise, with early evidence from Sweden showing positive results for off-patent intravenous fosfomycin^50^. The Joint Action on Antimicrobial Resistance and Healthcare-Associated Infections (EU-JAMRAI) has outlined a mechanism for a partially delinked subscription model at the EU level^41^, and other mechanisms have also been proposed^51^. This would be especially relevant for small markets, such as paediatric formulations of narrow-spectrum antibiotics, which may never reach sufficient volumes to be financially attractive. Political will to consider and agree to the exact size of the revenue guarantee, and differential ability of member states to pay would be crucial for the initiation and success of such collaborative models^51^. Estimating the exact size of revenue guarantee for each antibiotic will also need to take into account several factors, including public health importance of pathogens targeted, costs involved in production, the extent of pre-existing supply, and the budget impact for countries involved^51^. Subscription style payment models need to be designed with terms and conditions to minimise potential risks and unintended consequences, such as MAHs not supplying the relevant antibiotic equitably in sufficient volumes to where it is needed, or the creation of incentives that negatively impact security of supply of other antibiotics^5^.

### Harmonising and stockpiling products

In several cases, antibiotic markets are fragmented, meaning that clinicians within different countries prescribe antibiotics at different strengths and formulations^32^. Harmonizing antibiotic strengths and formulations across Europe would simplify manufacturing and improve supply predictability. A formalised exchange between countries to identify and compare available antibiotic strengths, formulations and package sizes would highlight harmonization opportunities. The EMA has already mapped paediatric doses and formulation of amoxicillin to identify opportunities for supply sharing across national borders^52^. Typically, harmonisation occurs when producers abandon less lucrative strengths and formulations. Proactively formalising such measures would require significant collaboration with national procurement agencies and generic manufacturers, both of which may be reluctant to share commercially sensitive information. Additionally, national antibiotic prescribing guidelines may require adjustments, necessitating significant stakeholder involvement and buy-in. Harmonised packaging and labelling would facilitate joint procurement efforts applicable to multiple member states and ensure that stockpiles of antibiotics could be deployed across Europe without re-packaging.

EU member states use national medicine stockpiling to address shortages^53^, which are most commonly short-term. The medicines, quantities and operational models of these stockpiles differ from country to country. Stockpiling does not resolve underlying market challenges and is generally considered expensive. However, from a solidarity perspective, pan-European stockpiling can prevent larger markets from crowding out smaller ones. Virtual stockpiling – an overview of decentralised supply buffers – has been recommended as a pan-European solution^54^. Existing platforms, such as the European Shortages Monitoring Platform (ESMP)^55^, or the European Medicines Verification System (EMVS)^56^, could be adapted for this purpose.

Virtual stockpiling could be combined with an increase in private sector mandatory inventories, providing the basis for balancing physical stockpiles across member states and determining the need for EU-coordinated procurement. Complementing this with public sector inventories would address country- or region-specific demand surges, and the rapid deployment of antibiotics where needed. HERA’s stockpiling strategy, currently under development, could also utilize resource reserves to protect EU residents from disasters and manage emerging risks^57^. Close collaboration with the European Centre for Disease Prevention and Control (ECDC) is vital to align actions with public health needs.

Making optimal use of stockpiling approaches is closely linked to improved monitoring of antibiotics at risk of shortage, whether due to demand shocks (e.g., large and sudden epidemics), or supply disruptions (e.g., manufacturing accidents or geopolitical issues). Given fragmented governance structures for antibiotic procurement, a lack of clarity exists within and between health systems regarding distribution of responsibility to determine which shortages pose sufficient risk to public health to warrant concerted action. This lends itself to increased dialogue at the EU level and aligns with the aspirations of the Pharmaceutical Strategy for Europe^58^. As of January 2022, the EMA mandate was expanded to include monitoring medicine shortages that might lead to crisis situations and reporting shortages of critical medicines during crises^59^. EMA and HERA closely monitor demand and supply of antibiotics and interact with MAHs for the timely detection of unexpected shortfalls^60^. Proposed revisions to EU pharmaceutical legislation include a provision that could include antibiotics in the EU’s list of critical medicines, even outside of emergency situations. This would increase flexibility for EU-level action, including stockpiling^61^. In October 2023, the Commission issued a communication on addressing medicines shortages, launching inter alia a “voluntary solidarity mechanism for medicines” in short supply, allowing for the redistribution of stocks among member states^61^. Precedent for this includes the Baltic Procurement Initiative which encompasses free-of-charge lending of medicines and medical devices in case of shortage^44^.

It is important to note that the impact of high-income country stockpiles on the availability of medicines in low- and middle-income countries has not been studied sufficiently. The recent experience of the global COVID-19 vaccine inequity highlights that stockpiles should be designed to avoid the creation of scarcity in less well-resourced countries^62^.

### Strengthening manufacturing capacity

Most disruptions in antibiotic supply can be attributed to the small number of actors involved and low prices (see above – purchasing arrangements), particularly in API manufacturing and conversion to medicines. Despite overall lack of transparency in raw material procurement and the location of reaction intermediary or API producers, it is clear that a substantial share of key ingredient production is outside the EU. For instance, it has been estimated that 45% of the API suppliers of antibiotics delivered to Sweden are from China (27%) or India (18%), with the remaining 55% spread across the world including a significant European presence (Italy, Spain and France alone account for 21%)^17^. These global dependencies may increase supply risks due to distance and geographic concentration, and to geopolitical factors that may be leveraged for critically important products such as antibiotics.

Despite European and national obligations for MAHs to notify relevant authorities of foreseeable temporary or permanent supply disruptions, a lack of transparency persists, partly due to sub-optimal information-sharing between the numerous stakeholders in the pharmaceutical supply chain^63,64^. Better understanding the antibiotics manufacturing and supply landscape is a prerequisite for strengthening essential structures and processes. However, supply chain information is often protected and difficult to obtain^54,65^. Counteracting this problem could begin with the creation of a database mapping the production capacity of all European factories in the antibiotics supply chain at the EU level, for example hosted by the EMA in collaboration with HERA. This would allow prompt identification of alternative producers when shortages occur and reduce supply risks by procuring from multiple sources^66^ and enable longer term planning for any “reshoring” efforts (see below). Indeed, increasing pharmaceutical supply chain transparency is highly desirable to facilitate API production and address shortages beyond antibiotics^13^. New Zealand has already implemented relevant policy^67^, with the Medicines and Medical Devices Safety Authority responsible for licensing medicine manufacturing sites and maintaining a publicly available list of the names and locations of API producers, finished product manufacturers, product sponsors and product MAHs.

Reinforcing diverse geographic manufacturing capacities would be advantageous to ensuring global supply security. For European production of antibiotics and APIs, this could be supported by a range of measures, including those discussed in previous sections. For example, innovative tendering procedures, such as those recently implemented in Germany^68^, can stipulate that contracts for antibiotics must have a minimum number of suppliers, including at least one (or more) using European API production. Measures to stimulate “reshoring”, i.e., the relocation of antibiotic manufacturing inside the EU (backshoring) or near the EU (nearshoring), include subsidies to private companies willing to relocate, joint public-private ownership of production plants, and even the extreme, but highly expensive solution of fully public manufacturing^17^.

EMA landscape analyses of antibiotics at high risk for shortage owing to sole or geographically concentrated producers, and leveraging information from other measures suggested earlier, could help steer manufacturing diversification efforts to the most needed ingredients, APIs or medicines.

Administrative and regulatory adjustments to facilitate manufacturer “reshoring” could be considered. However, it would be important to link support measures to conditions for improved transparency and for social and environmental responsibility. Linked to other measures, such as national API buffer stocks, additional actors could be mandated to produce antibiotics. For example, both Austria and Switzerland, which both have traditions of producing magistral preparations in community pharmacies, increasingly produced antibiotic magistral preparations during the 2023–2024 winter season^53^. However, this is less feasible in EU countries in which community pharmacy magistral preparations are not commonplace, and would in any case require sufficient supply of the necessary APIs. The ultimate focus should be eliminating reliance on sole suppliers of important antibiotics, regardless of the location of those suppliers.

In its Pharmaceutical Strategy for Europe, the European Commission committed to “promote investment and coordinate research, development, manufacturing, deployment and use for novel antibiotics as part of the new EU Health Emergency Response Authority”^58,69^. Such coordinated action is also necessary for existing antibiotics. However, existing initiatives focused on strengthening antibiotic production in Europe still occur predominantly at the national level. This is exemplified by recent investment by the Austrian government and the pharmaceutical company Sandoz to extend penicillin manufacturing capacity^70^, and the creation of financial incentives by the French government to encourage domestic medicine manufacturing^13^. It is evident that strengthening manufacturing capacity requires long-term solutions to supply challenges, requiring substantial investment and meticulous planning. Moreover, an end-to-end approach that addresses ingredient procurement, API manufacturing and final products is necessary. Public investment to strengthen manufacturing capacity could be delivered through subsidies or through public ownership of certain generic manufacturers^13^. However, the implications for competition in the common market and the limitations imposed by state aid rules would need to be carefully considered. The Critical Medicines Alliance hosted by HERA can be leveraged to focus and refine future actions for antibiotics^71^.

## Conclusion

Ensuring reliable access to effective antibiotics is crucial for improving patient outcomes, both now and in the future. While multiple antibiotics are generally available in Europe to treat common infections, maintaining the efficacy of those antibiotics for future generations requires that the most appropriate antibiotic is available when needed. The policy options presented in this article seek to support these efforts by improving commercial attractiveness, better understanding product availability, supply chain structures and vulnerabilities, and strengthening structures and processes. These policies are complex and affect or involve diverse stakeholders in healthcare, industry, public agencies and academia. Therefore, multisectoral collaboration is necessary to enable integrated approaches that combine multiple policies and concerted efforts to ensure access to existing antibiotics.

In September 2024, the President of the European Commission set out the development of a Critical Medicines Act to address the issue of shortages as the first order of business in her letter to the incoming Commissioner for Health, building on the Conclusions of the Belgian Presidency of the Council of the EU^72^. Several of the options proposed in this article could be considered in the development of such a regulatory framework. The letter also reinforces the importance of tackling AMR, strengthening the potential for a special focus on antibiotics in this context. In the global context, decision-makers could build on the political declaration on AMR adopted by the United Nations General Assembly in September 2024 to explore relevant measures beyond the EU^73^.

Indeed, despite focusing on the EU/EEA setting, these issues and policy options have global applicability. It is important to note that this paper does not examine the potential unintended consequences of suggested options in detail; for instance, some of the measures suggested here might facilitate redistribution of available products away from some markets outside the EU, exacerbating existing challenges there. The EU has a strong global voice and should leverage it towards ensuring sustained access to existing antibiotics both within and beyond its borders.
